# Supplementary material for: Experimental Quantum-enhanced Cryptographic Remote Control
Source: Sci Rep. 2019 Apr 9;9:5809. doi: 10.1038/s41598-019-42278-8 (PMC6456578; doi:10.1038/s41598-019-42278-8)
Supplement: Supplementary file 1 — Supplementary Materials: Experimental Quantum-enhanced Cryptographic Remote Control [file 41598_2019_42278_MOESM1_ESM.pdf]

Supplementary Materials: Experimental Quantum-enhanced Cryptographic Remote Control

Xiao-Ling Pang,[1,2] Lu-Feng Qiao,[1,2] Ke Sun,[1,3] Yu Liu,[1,3] Ai-lin Yang,[1,2] and Xian-Min Jin[1,2,*]

[1]State Key Laboratory of Advanced Optical Communication Systems and Networks, School of Physics and Astronomy, Shanghai Jiao Tong University, Shanghai 200240, China
[2]Synergetic Innovation Center of Quantum Information and Quantum Physics, University of Science and Technology of China, Hefei, Anhui 230026, China
[3]Zhiyuan Innovative Research Center, Shanghai Jiao Tong University, Shanghai 200240, China

Commands reliability testing

To test the reliability of cryptographic remote control scheme, commands emitted by the UAV controller are intercepted and processed by another microcontroller (STM32). As for general cases, without encryption, the five-time repeated commands intercepted are demonstrated in FIG. S1(a). Inserted is the command structure which consists of 32 bytes, and each byte consists of eight “0” or “1” bits. Thus the value of a byte ranges from 0 to 255.

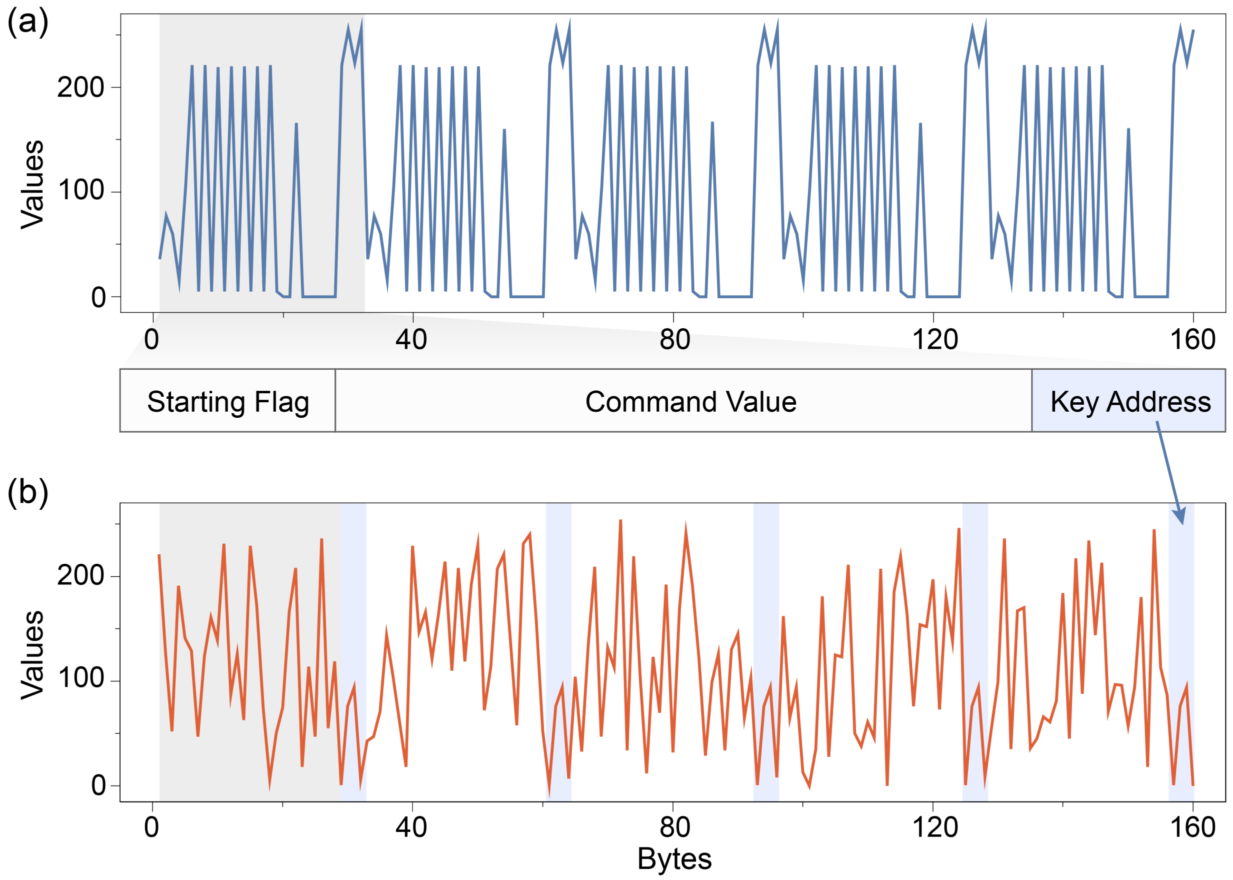

FIG. S1: **Intercepted commands before and after encryption.** **(a)** The plaintexts of five-time repeated commands. The inserted represents the structure of a command. **(b)** The ciphertexts of five-time repeated commands. Shadows mark the key information in each commands.

*Electronic address: xianmin.jin@sjtu.edu.cn

TABLE I: **Intercepted values of plaintexts sent by the UAV controller. Red values show the difference.**

**Connection**	36	77	60	16	105	221	5	221	5	219	5	220	5	220	5	220
	5	221	5	0	0	166	0	0	0	0	0	0	221	255	223	255
**Backward**	36	77	60	16	105	221	5	221	5	219	5	232	3	221	5	220
	5	221	5	0	0	149	0	0	0	0	0	0	221	191	215	255
**Turn Left**	36	77	60	16	105	221	5	220	5	234	3	219	5	220	5	220
	5	221	5	0	0	168	0	0	0	0	0	0	221	255	215	255
**Turn Right**	36	77	60	16	105	221	5	221	5	208	7	219	5	220	5	221
	5	220	5	0	0	168	0	0	0	0	0	0	221	255	215	255
**Forward**	36	77	60	16	105	220	5	220	5	220	5	208	7	220	5	219
	5	221	5	0	0	168	0	0	0	0	0	0	221	255	215	255

The key address information added in the command is not encrypted, which contains no information without local quantum key storage devices.

As for situations with quantum-enhanced encryption, commands sent by the UAV controller are encrypted by one-time pad algorithm. The ciphertexts intercepted are shown in FIG. S1(b). We can see that the five-time repeated commands has been brought into chaos, except for the bytes which carry key address information (marked in shadows). According to the key address information, the controllee can read out quantum keys and perform one-time pad decryption process. The randomness of ciphertexts has been tested and presented in Figure 3 and Table I in the main text.

A brief structure of a command is shown in the insert of FIG. S1. Even different functional commands share parts of the same bytes to maintain stable communication process. Therefore, it is not necessary to encrypt all 32 bytes in a command, and we can just perform one-time pad encryption on the bytes that carry fatal information, to save quantum keys. Values of plaintexts of five commands (blue lines) presented in Figure 4 in the main text are shown in Table I. According to the command structure, they are similar but not the same.
